# Catheter Ablation of His-Purkinje System–Related Ventricular Tachycardia After Left Bundle Branch Area Pacing

**DOI:** 10.1016/j.jaccas.2025.106711

**Published:** 2026-01-13

**Authors:** Yu Zhang, Weifeng Jiang, Xu Liu, Xiaofeng Hu

**Affiliations:** Department of Cardiology, Shanghai Chest Hospital, Shanghai Jiao Tong University School of Medicine, Shanghai, China

**Keywords:** catheter ablation, His-Purkinje system related VT, left bundle branch area pacing

## Abstract

**Background:**

Sustained His-Purkinje system–related ventricular tachycardia (HPS-VT) after left bundle branch area pacing (LBBAP) has not to our knowledge been previously reported.

**Case Summary:**

A 65-year-old woman presented 1 month after dual-chamber LBBAP with hypotension and recurrent palpitations. Electrocardiogram showed 2 bundle branch block–like VT morphologies; device interrogation demonstrated ventriculoatrial dissociation, consistent with HPS-VT. Intracardiac echocardiography localized the left bundle branch pacing lead to the anterior left ventricular septum. Targeted septal ablation terminated VT, but early recurrence required a second ablation, resulting in complete atrioventricular block; pacing was revised to the right ventricular septum. VT had not recurred at the 1-month follow-up.

**Discussion:**

This case illustrates that HPS-VT can complicate LBBAP. Lead-related conduction system stimulation or injury may provide the arrhythmic substrate. Diagnosis and management are challenging, requiring mechanistic understanding to guide therapy.

**Take-Home Message:**

Sustained HPS-VT may occur after LBBAP; careful electrophysiologic assessment and accurate diagnosis are essential to minimize conduction system injury.

## Visual Summary

**Figure:**
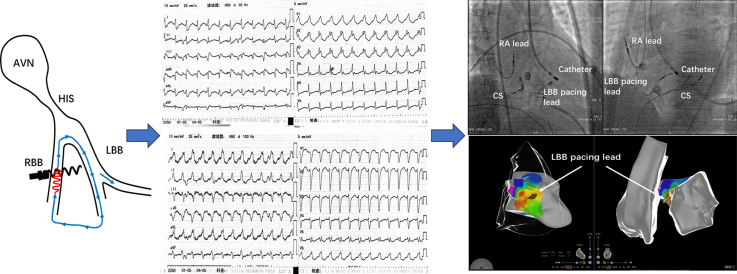

## History of Presentation

A 65-year-old woman presented with recurrent palpitations for 1 month and re-exacerbation with dizziness for 1 day. On arrival, her blood pressure was 90/60 mm Hg, and she reported presyncope. Previous electrocardiograms had demonstrated a right bundle branch block pattern.

**Take-Home Messages**

• Sustained His-Purkinje system–related ventricular tachycardia may occur after left bundle branch area pacing.
• Careful electrophysiologic assessment and accurate diagnosis are essential to minimize conduction system injury.

## Past Medical History

The patient had a history of sick sinus syndrome, and she had undergone dual-chamber pacemaker implantation with left bundle branch area pacing (LBBAP) 1 month earlier. She had no known hypertension, diabetes, or coronary disease, and no drug allergies. There was no family history of sudden death.

## Differential Diagnosis

Considerations included supraventricular tachycardia with aberrancy or atrioventricular re-entry, bundle branch re-entrant VT (BBRVT), and His-Purkinje system (HPS)–related ventricular tachycardia (VT) (fascicular/interfascicular).

## Investigations

**Figure 1:**
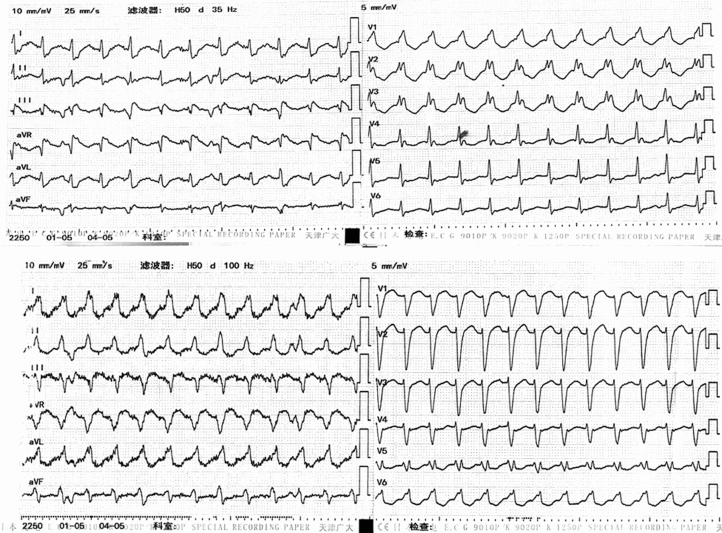
Emergency 12-Lead ECGs Showing 2 VT Morphologies Two bundle branch block–like VT morphologies with similar cycle lengths are demonstrated. In lead V1, one tracing shows an RBBB-like pattern and the other an LBBB-like pattern. LBBB = left bundle branch block; RBBB = right bundle branch block; VT = ventricular tachycardia.

Twelve-lead electrocardiograms demonstrated 2 bundle branch block–like VT morphologies with similar ventricular rates (Figure 1). Device interrogation indicated ventriculoatrial dissociation (V:A = 3:1), supporting VT. Baseline laboratory results and transthoracic echocardiography were unremarkable.

## Management

### First procedure (index admission)

**Figure 2:**
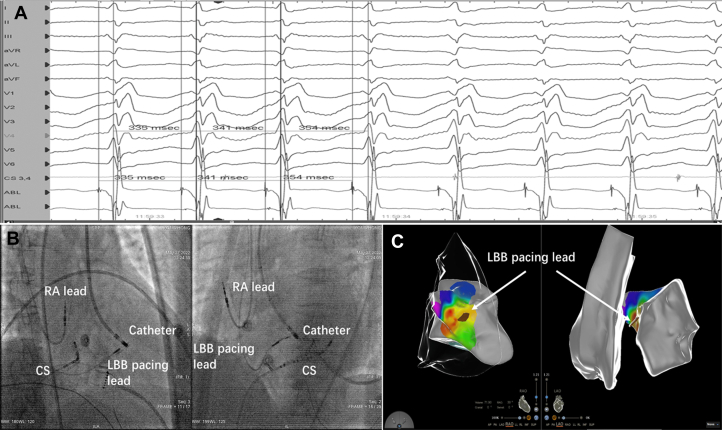
Integrated Electrophysiological and Imaging Findings During VT Mapping and Ablation (A) High-gain intracardiac electrograms during VT showing Purkinje-Purkinje (PP) interval changes preceding RR changes, a diagnostic feature of HPS-related VT. (B) Fluoroscopy in the right anterior oblique and left anterior oblique projections depicting intracardiac catheter positions and the LBBAP lead traversing the interventricular septum. (C) Three-dimensional activation map integrated with intracardiac echocardiography shell shows the earliest LV anterior septal activation immediately adjacent to the LBBP lead marker. CS = coronary sinus; HPS = His-Purkinje system; LBB = left bundle branch; LBBAP = left bundle branch area pacing; LBBP = left bundle branch pacing; LV = left ventricular; RA = right atrial; VT = ventricular tachycardia.

During VT, Purkinje-Purkinje (PP) interval changes preceded RR, and premature beats advanced the His potential with reset, which was diagnostic of HPS-related VT (Figure 2A). Intraprocedural intracardiac echocardiography showed the LBBAP lead on the anterior left ventricular (LV) septum, immediately adjacent to the earliest endocardial activation on 3-dimensional mapping (Figures 2B and 2C). Targeted LV septal endocardial ablation opposite the lead promptly terminated VT and rendered it noninducible, where a sharp left bundle branch potential was recorded preceding the local ventricular electrogram.

### Early recurrence and second procedure (postprocedure day 1)

**Figure 3:**
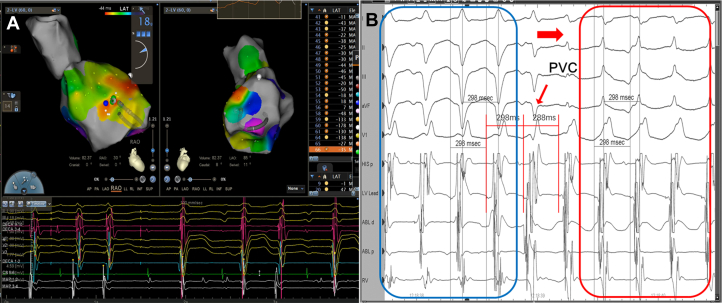
3-Dimensional Activation Mapping and Electrophysiological Evidence of Distal HPS-Related VT During the Second Procedure (A) Second-procedure 3-dimensional activation map depicting earliest activation on the septal surface. (B) During mapping, a fusion premature ventricular complex advances the His potential and resets the tachycardia, while the QRS morphology shifts from an LAFB-like pattern (blue box) to an LPFB-like pattern (red box), consistent with a distal (interfascicular) re-entry mechanism. HPS = His-Purkinje system; LAFB = left anterior fascicular block; LPFB = left posterior fascicular block; PVC = premature ventricular complex; VT = ventricular tachycardia.

During the second procedure, the VT was paroxysmal and nonsustained. Connecting the left bundle branch lead to a multichannel recorder confirmed early bundle electrograms and persistent PP-to-RR precedence. The earliest activation site remained located at the LV anterior septal region (Figure 3A). A fusion premature ventricular complex advanced the His potential, indicating a re-entrant mechanism. VT morphology switched between left anterior fascicular block–like and left posterior fascicular block–like without cycle-length change, favoring distal (interfascicular) re-entry over BBRVT (Figure 3B). Repeat focal ablation at the anterior LV septum terminated VT but resulted in complete atrioventricular block; the pacing lead was subsequently repositioned to the right ventricular septum, with stable pacing parameters achieved.

## Outcome and Follow-Up

No VT recurred on in-hospital monitoring or 1-month ambulatory follow-up. Symptoms resolved completely, and pacing thresholds and sensing remained stable.

## Discussion

To our knowledge, sustained HPS-related VT after LBBAP has not been previously reported. This case suggests that, in selected patients with underlying HPS disease, physiologic pacing in close proximity to the conduction system may facilitate sustained VT. A plausible substrate is either HPS injury from the left bundle branch lead—predisposing to BBRVT—or direct lead-mediated stimulation of the HPS.^1^ Because right-ventricular entrainment was not performed, the mechanism could not be definitively established; ablation was undertaken and resulted in complete atrioventricular block.^2^ Had classic BBRVT been confirmed, right bundle ablation would likely have been a more targeted strategy, particularly given the patient's preexisting complete right bundle branch block.^3^ Although the first ablation terminated VT, widespread HPS disease likely permitted distal Purkinje re-entry, accounting for recurrence and the need for a second intervention. A key lesson is that left bundle branch pacing may be followed by HPS-related re-entrant VT, mandating meticulous diagnostic confirmation and mechanistic differentiation before ablation. Taken together, this first documented case demonstrates that left bundle branch pacing can, in rare instances, be followed by sustained HPS-related VT and that both diagnosis and therapy are challenging; these observations may inform clinical decision-making and procedural planning in similar scenarios.

**Visual Summary:**
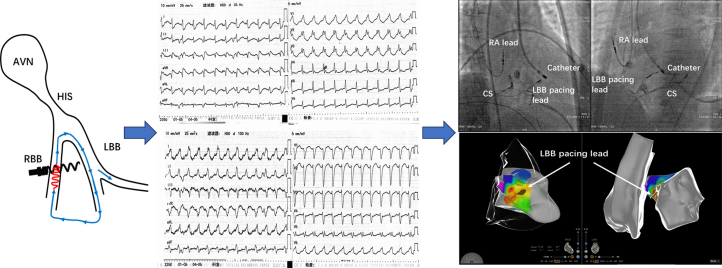
Illustration of His-Purkinje System–Related Ventricular Tachycardia After Left Bundle Branch Area Pacing, Showing Successful Ablation But Also Underscoring Important Limitations in the Diagnostic and Procedural Approach AVN = atrioventricular node; CS = coronary sinus; LBB = left bundle branch; RA = right atrium; RBB = right bundle branch.

## Conclusions

This first documented case shows that sustained HPS-related VT may occur after LBBAP. Careful diagnostic confirmation and individualized ablation are required, given the risk of conduction injury and pacing revision.

## Funding Support and Author Disclosures

This research was supported by the Noncommunicable Chronic Diseases-National Science and Technology Major Project (2024ZD0521906). The authors have reported that they have no relationships relevant to the contents of this paper to disclose.
